# Association between participation in the Northern Finland Birth Cohorts and cardiometabolic disorders

**DOI:** 10.1080/07853890.2023.2186478

**Published:** 2023-03-22

**Authors:** Martta Kerkelä, Mika Gissler, Tanja Nordström, Olavi Ukkola, Juha Veijola

**Affiliations:** a Research Unit of Clinical Medicine, University of Oulu, Oulu, Finland; b Department of Knowledge Brokers, Finnish Institute for Health and Welfare, Helsinki, Finland; c Research Centre for Child Psychiatry, University of Turku, Turku, Finland; d Academic Primary Health Care Centre, Region Stockholm, Sweden; e Department of Molecular Medicine and Surgery, Karolinska Institute, Stockholm, Sweden; f Northern Finland Birth Cohorts, Arctic Biobank, Infrastructure for Population Studies, Faculty of Medicine, University of Oulu, Oulu, Finland; g Research Unit of Population Health, University of Oulu, Oulu, Finland; h Medical Research Center, University Hospital and University of Oulu, Oulu, Finland; i Research Unit of Internal Medicine, University of Oulu, Oulu, Finland; j Department of Psychiatry, University Hospital of Oulu, Oulu, Finland

**Keywords:** Cardiometabolic disorders, follow-up study, Birth cohorts

## Abstract

**Background:**

We studied the association between participation in the longitudinal follow-up study and cardiometabolic disorders in two longitudinal studies which started prospectively in the antenatal period: the Northern Finland Cohort 1966 (NFBC1966) and the Northern Finland Birth Cohort 1986 (NFBC1986). Both birth cohorts have been followed up since birth with multiple follow-ups including questionnaires, and clinical examinations.

**Methods:**

The NFBC studies were compared to comparison cohorts of individuals who were born in the same area as the study cohorts, but in different years. The data for the comparison cohort were obtained from registers. The cumulative incidence rates of hospital-treated cardiometabolic disorders were calculated for study and comparison cohorts covering the age of 7–50 years in NFBC1966 and the age of 0–29 years in NFBC1986. Cardiometabolic-related causes of death were analysed in NFBC1966 and the comparison cohort from the age of 0–50 years. The analysed cardiometabolic disorders were diabetes mellitus, coronary artery disease, hyperlipidaemia, obesity, hypertension, and cerebrovascular disorders. The risk ratio (RR) with 95% confidence intervals (CI) was calculated by sex.

**Results:**

In NFBC1966, no differences in cumulative incidences of cardiometabolic disorders or cardiometabolic-related deaths compared to the comparison cohort were found. Male members of NFBC1986 had decreased risk of obesity (RR: 0.45, 95% CI: 0.27–0.75) and any cardiometabolic disorders (RR: 0.75, 95% CI: 0.59–0.95) compared to the comparison cohort.

**Conclusions:**

The results suggest that participation in the NFBC1986 may have a weak positive health effect among men. Agreement to follow-up studies focusing on diet, substance use, and physical activity, may slightly decrease the incident risk of cardiometabolic disorders in the study population.

**KEY MESSAGES**
- Even mild interventions, such as follow-up studies in the prospective follow-up studies, might affect participants’ behaviour and consequently the incidence of cardiometabolic disorders
- The fact that follow-up itself might affect the study population in terms of risk factors, has to be taken into account when estimating the representativeness of the followed population.

## 1. Introduction

In prospective population-based cohort studies, a defined population is selected for longitudinal assessment of exposure-outcome relations. Data collection procedures include clinical examinations, questionnaires, tests, interviews, or linkage to existing data [1]. The Hawthorne effect is a phenomenon that occurs when the mere act of being observed in a study changes the behaviour of the study’s participants. This can impact the validity of the study’s results, as the behaviour that is being observed may not be representative of the participants’ normal behaviour [2]. In the Derbyshire Smoking Study, the Hawthorne effect was observed in a group of approximately 6,000 adolescents whose smoking habits were surveyed with a yearly questionnaire from 1974 to 1978 in selected schools. The findings showed that the prevalence of smoking was lower in schools that had been surveyed for five years [3]. A meta-analysis of 15 studies published between 2012 and 2022 found that the Hawthorne effect is common in medical research, which can limit the study’s external validity and result in gaps in medical knowledge [4]. Nevertheless, whether the intensive follow-ups affect the study population has not been thoroughly studied. The follow-up studies conducted may have a wide range of effects on the target population, even though the purpose of the prospective birth cohort setting is not to intervene but to get a representative sample of the population.

Cardiovascular disorders are the leading cause of death globally, and a proportion are preventable through healthy lifestyle choices. Major risk factors for cardiovascular disorders are tobacco use, unhealthy diet and obesity, physical inactivity, and harmful use of alcohol [5]. One successful intervention in encouraging a healthy lifestyle was the Stanford Community study. The aim was to reduce fat content in the daily diet and the health information was broadcast *via* the mass media. After two years of intervention, a significant drop in the population’s negative health factors (cholesterol level, blood pressure, smoking rate) could be seen [6]. Another well-known population-level health intervention is the North Karelia project, in which the aim was to reduce cardiovascular risk factors through interventions including broad health promotion and policies in the 1970s onwards. The project was successful: the results indicated substantial and positive shifts in health-related behaviours, the community-based part of the project ‘hypertension control programme’ brought hypertension under control for a significant proportion of hypertensive individuals, and over the years, the coronary heart disease mortality rates decreased [7]. The North Karelia project and Stanford Community study powerfully demonstrate the effect of population-based intervention in cardiometabolic disorders.

We were able to study the association between participation in a longitudinal follow-up study and cardiometabolic disorders in two longitudinal studies which started prospectively in the antenatal period: namely the Northern Finland Cohort 1966 (NFBC1966) [8] and the Northern Finland Birth Cohort 1986 (NFBC1986) [9]. Since birth, both birth cohorts have undergone several follow-ups such as surveys and clinical examinations. Even though the follow-ups of the NFBC studies were not supposed to be interventions, many of them included questions and screenings on lifestyle, exercise, diet, and use of intoxicants. Several follow-ups in the NFBC studies also included clinical examinations, in which weight and height, blood pressure, physical activity, and numerous other clinical measurements were measured. The hypothesis is that the members of NFBC studies might live more healthily due to follow-up and therefore have fewer cardiometabolic disorders than comparison cohorts.

## 2. Materials and methods

### 2.1. Cohorts

The NFBC1966 covers people whose expected date of birth was in 1966 in the former two northernmost provinces of Finland, Oulu, and Lapland. The cohort included 12,055 mothers and 12,231 children. Of the NFBC1966 members, 189 (1.5%) were born in 1965, 11,999 (97.9%) in 1966 and 63 (0.5%) in 1967. NFBC1966 is a longitudinal and prospective birth cohort with several follow-ups. The comparison cohort for the NFBC1966 comprises all liveborn children in the provinces of Lapland and Oulu in the years 1965 and 1967. The cohort included 24,471 participants, 12,465 (50.9%) born in 1965 and 12,006 (49.1%) born in 1967. All data for the comparison cohort was collected from Finnish registers: personal identification numbers were obtained from the Digital and Population Data Services Agency and medical histories were obtained from the Finnish Institute of Health and Welfare (THL).

The NFBC1986 covers people with an expected date of birth between 1 July 1985 and 30 June 1986 in the former Finnish provinces of Oulu and Lapland. The cohort consisted of 9,362 mothers and 9,479 children. The NFBC1986 is a prospective, longitudinal birth cohort with numerous follow-ups. The NFBC1986 comparison cohort consists of all people born in the provinces of Lapland and Oulu in 1987. A sub-sample from the register-based Finnish Birth Cohort 1987 (FBC 1987) was used as the comparison cohort. All children born in Finland in 1987 who survived the perinatal period and were alive at seven days after birth are included in the FBC 1987 study, with all data collected from several registers. Members of FBC 1987 have never been contacted [10]. Information on the place of birth was obtained from the Medical Birth Register, which is currently maintained by the Finnish Institute for Health and Welfare (THL).

### 2.2. Follow-ups in NFBC1966

The first follow-up for the NFBC1966 cohort was conducted during the mother’s pregnancy. The follow-up included a questionnaire (including questions about background, life situation, and living habits) during pregnancy (from the 24th to the 28th gestational week) and delivery information. The data were collected by the midwives at antenatal clinics [11]. The second follow-up was conducted at age 1 year, including a questionnaire concerning children’s growth, health, and development, with a 91.2% participation rate [12]. The next follow-up was at age 14 years, with a participation rate of 93.6%. The follow-up was conducted with a postal questionnaire, including questions for NFBC1966 members about their growth and health, physical exercise, substance use (smoking, alcohol, and other intoxicants), living habits, school performance, and family situation [13]. The questions regarding physical exercise, health and substance use are described in more detail in Supplement Table 1.

The next follow-up for the whole cohort was conducted at age 31 years. The follow-up included a postal questionnaire (participation rate 77.4%), including questions about a life situation, background information, exercise, physical performance (how often and how much), occupation, living environment, health (e.g. height, weight, history of cardiometabolic disorders, use of medications), diet, and living habits (questions on smoking, alcohol, and other intoxicant use) (Supplement Table 2). Clinical examination included a skin prick allergy test, physical performance tests (step test, grip strength test, back endurance test), blood pressure and pulse, blood tests, other laboratory tests (cholesterol, glucose, insulin, triglyceride), and spirometry (participation rate 71.3%) [14].

The latest follow-up has been conducted at age 46 years, with a 66.5% participation rate in the questionnaire and 56.7% in the clinical examination. The questionnaire included questions about background, lifestyle (questions about smoking, alcohol, and other intoxicant use), health (e.g. disorders and medications), economy, work, and mental resources (Supplement Table 3). In the clinical examination, data were collected on cardiovascular health (brachial pressure, blood pressure, 15-led rest ECG, echo and carotid ultrasound), allergies (skin allergy test, spirometry, whole body examination by dermatologist), eyes (eye examination), physical activity and fitness (two-week accelerometer measurements, back muscle strength test, step test, hand grip strength test), pain perception and tolerance (pressure pain and thermal perception), musculoskeletal health (spine, ankle, and lower back movement tests, knee, ankle foot radiography and lumbar MRI), height, weight, waist and hip circumferences, dental health, cognitive tests, and biological samples [15].

The follow-up in NFBC1966 also includes several subsamples. One of the subsamples focusing on the health and life satisfaction of male cohort members was conducted on a random subsample of males at age 24 years (*N* = 2,500) [16]. The follow-up consisted of a questionnaire including questions about physical activity, diet, smoking habits, overall health, and relationships. Another sub-sample focusing on health was conducted on a random subsample of 31-year-old follow-up participants (*N* = 196). The follow-up consisted of exercise and food diaries for 7 days [17]. Further details of all substudies conducted in NFBC1966 are provided in Supplement Table 4.

### 2.3. Follow-ups in NFBC1986

The NFBC1986 data collection began throughout the mother’s pregnancy. At antenatal clinics, mothers were given three questionnaires that included questions on their background, smoking and alcohol use, general health, fatigue throughout pregnancy, and delivery information [18].

At age 7 years, the second follow-up was completed in the autumn of the first school year. The questionnaire, which included information about children’s growth, development, and health, as well as their socioeconomic factors, was sent to parents (participation rate 90%) [19]. The next follow-up was scheduled for the spring of the children’s first school year. The follow-up included two questionnaires for parents, one for themselves and one for the children’s teachers. With a participation rate of 90%, the parents’ questionnaire included a modified (one of the internalizing items was modified) Rutter A2 [20]. The teachers completed Rutter B2 [21], with a 92% participation rate. Rutter scales assess children’s behavioural and emotional characteristics.

The next follow-up was undertaken at the age of 15–16 years, with a questionnaire for adolescents (participation rate 77.9%) and parents (74.5%). The adolescent’s questionnaire included questions about family, school, health (history of heart diseases, weight and height, and blood pressure), physical activity (e.g. times and duration of physical exercise), sexual behaviour, substance use (smoking, alcohol, or other intoxicants), diet (eating habits, use of sugar and fat, amount and times of eating bread, dairy products, sweets, etc.), living habits and hobbies. The questions regarding physical exercise, diet, cardiovascular health, and substance use are described in Supplement Table 5. The adolescents were also invited to clinical examinations (participation rate of 73.5%). The clinical examination included weight, height, waist-hip measurements, sitting height, spirometry, blood pressure, pulse rate, blood samples, physical activity (bicycle ergometer) test, prick tests, and questions about puberty, nutrition, smoking, and use of alcohol.

NFBC1986 also includes multiple sub-studies. At age 18 years, a questionnaire including questions about low back pain history, medical history, quality of life, nutrition, socioeconomic status, leisure activities, history of injuries, smoking history, occupational exposure, sports activities, and psychological factors, was sent to cohort members living in Oulu and the surrounding municipalities [22]. The participants of the substudy were also invited to an MRI scan of the lumbar spine at age 19–22 years and 29–32 years [23]. At age 24 years, the ESTER- Preterm Birth, Pregnancy and Offspring Health in Adult Life study was conducted on a subpopulation of NFBC1986. The data collection consisted of blood sampling, blood pressure, BMI, waist, and hip measurements [24]. The gynaecological health of young females was studied by a questionnaire sent to female members of NFBC1986 at age 26 years. The questionnaire included questions on socio-demographic and health background, mainly about reproduction, menstruation, and infertility [25]. Further details of all substudies conducted in NFBC1986 are provided in Supplement Table 6.

### 2.4. Final datasets of NFBC and comparison cohorts

From NFBC1966 we excluded those who were born in 1965 (*N* = 186) or 1967 (*N* = 63) and stillbirths (*N* = 153), while the comparison cohort comprises liveborn children in the years 1965 and 1967. The final dataset of NFBC1966 comprised 11,723 participants, 6,000 males and 5,723 females. The comparison dataset comprised 24,471 participants, 12,511 males and 11,960 females.

To make the study and comparison cohorts comparable, from NFBC1986, we excluded those who died during the perinatal period (stillbirths *N* = 47, died during the first seven days of life *N* = 36). The final NFBC1986 dataset included 9,396 participants, 4,839 males, and 4,557 females. The comparison dataset included 8,959 participants, 4,550 males, and 4,409 females.

The Finnish register data were given for this specific study, and the data cannot be shared without authorisation from the register keepers and the University of Oulu. In the use of data, we follow the EU General Data Protection Regulation (679/2016) and Finnish Data Protection Act.

### 2.5. Cardiometabolic disorders

The following cardiometabolic disorders were considered as an outcome measure: Diabetes mellitus (differentiation into type 1, type 2 and unspecific types in ICD-9 and ICD-10); Coronary artery disease; hyperlipidaemia; overweight, obesity and other hyperalimentation; hypertension; cerebrovascular disorder, and any aforementioned cardiometabolic disorder. The Care Register for Health Care (CRHC), maintained by the Finnish Institute for Health and Welfare, was used to identify patients who had a diagnosis (primary or secondary diagnosis) of an above-described cardiometabolic disorder. We used the ICD-8 (1972–1986), ICD-9 (1987–1995) and ICD-10 (1996–2017) classifications (Table 1). The CRHC is one of the oldest individual-level hospital discharge registers and contains nationwide hospital discharge information on inpatient visits starting from 1967. From 1998 onwards, the register also includes information on specialised outpatient care in public hospitals. By law, all hospitals are obligated to report all inpatient care. The data covers public hospitals only. Several studies indicate that the quality of CRHC is high [26]. NFBC1966 and its comparison cohort were followed from age 7 to 50 years (1972–2017), while the information on the CRHC was incomplete before 1972. In NFBC1986 and its comparison cohort, the follow-up covered age 0 to 29 years (1985–2016). In addition, we examined cardiometabolic-related deaths in NFBC1966. Cardiometabolic-related deaths were defined with the corresponding ICD codes (Table 1) from statistical underlying cause-of-death diagnoses. The cause-of-death codes were obtained from the Cause of Death Register, maintained by Statistics Finland.

**Table 1.** List of cardiometabolic disorders and their ICD-codes.

	ICD-8 1969–1986	ICD-9 1987–1995	ICD-10 1996–2017
Diabetes mellitus	250	250	E10-E14
Type 1		2500B-2508B	E10
Type 2		2500A-2508A	E11
Unspecific type		2500C-2508C, 2500X-2508X	E12-E14
Coronary artery disease	410–414	410–414	I20–I25
Hyperlipidaemia	272	272	E78
Overweight, obesity, and other hyperalimentation	277.99	278	E65-E68
Hypertension	400–404	401–405	I10–I15
Cerebrovascular disorders	430–438	430–438	I60–I69
Any cardiometabolic disorder	250, 272, 277.99, 400–404, 410–414, 430–438	250, 272, 278, 401–405, 410–414, 430–438	E10-E14, E65-E68, E78, I10-I15, I20-I25, I60-I69

### 2.6. Statistical analysis

The cumulative incidence rates of cardiometabolic disorders in all hospital-treated cardiovascular disorders (including inpatient and specialized outpatient visits) were calculated for the study and comparison cohorts covering the full follow-up (age 7 to 50 years in NFBC1966; age 0 to 29 years in NFBC1986). Different types of diabetes mellitus were examined from 1987 onwards (age 2 to 29 years in NFBC1986 and age 22 to 50 years in NFBC1966). Due to the small number of cases of hyperlipidaemia and coronary artery disorders in the younger population (follow-up ends at age 29 years), the separate diagnosis classes are not included in the analysis. Risk ratios (RRs) with 95% confidence intervals (CIs) were calculated by sex separately in each diagnosis group. The age of the first onset of cardiometabolic diagnosis (median with IQR) is reported in each diagnosis group. The difference between the medians is estimated using quantile estimation (QE) and Q with *p*-values are reported [27]. The age of the first onset of cardiometabolic disorders was plotted over the full follow-up period in both NFBCs, separated by sex. Cumulative incidences of cardiometabolic-related causes of death were calculated to NFBC1966 and comparison cohorts at age 0 to 50 years. The age of death caused by any cardiometabolic disorders (median with IQR) by sex is also reported. Analysis was performed using R version 1.4.1106.

## 3. Results

**Figure 1.**
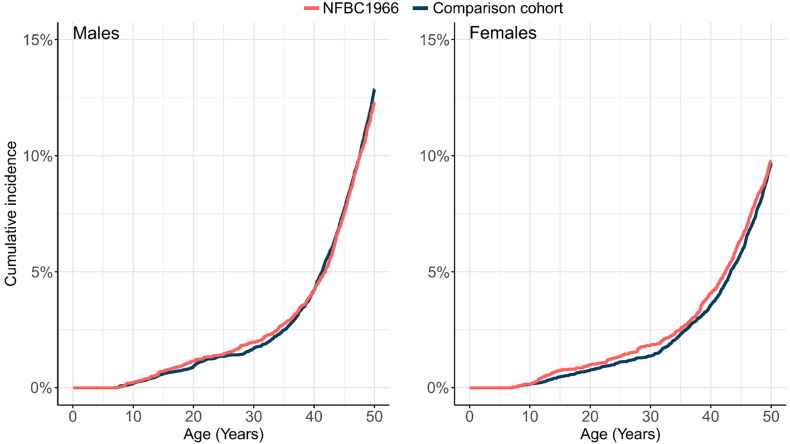
Cumulative incidence of first cardiometabolic disorders in NFBC1966 and comparison cohort at age 7 to 50 years by sex.

Table 2 reports the cumulative incidences % (*N*), Pearson’s chi-square statistics, and risk ratios (RR) with 95% confidence intervals (CI) of all hospital-treated cardiovascular disorders of NFBC1966 with the comparison cohort at age 7 to 50 years. No significant differences in cumulative incidences between NFBC1966 and the comparison cohort were found for cardiometabolic disorders. Female members of NFBC1966 had a lower age of onset of coronary artery disease (median age of onset 42.5 years vs 45.8 years; Q:3.85, *p* = 0.0.049), diabetes mellitus (median age of onset 38.1 years vs 41.7 years; Q:8.28, *p* = 0.004) and any cardiometabolic disorder (median age of onset 42.1 years vs 43.1 years; Q:4.16, *p* = 0.041) compared to the comparison cohort (Figure 1, Table 3). No difference in cumulative incidences or age of onset in different types of diabetes mellitus from age 22 to 50 years between NFBC1966 and the comparison cohort was found (Table 4).

**Table 2.** Cardiovascular disorders in NFBC1966 with a comparison cohort at age 7 to 50 years.

Diagnostic group	NFBC 1966	Comparison cohort	χ2	*p* Value	RR (95% CI)	*p* Value
Males	*N* = 6000	*N* = 12,511				
Cerebrovascular disorders	2.1 (127)	2.3 (286)	0.46	0.498	0.93 (0.75–1.14)	0.465
Coronary artery disease	2.8 (165)	2.9 (368)	0.47	0.495	0.93 (0.78–1.12)	0.466
Diabetes mellitus	3.5 (213)	3.6 (451)	0.02	0.884	0.98 (0.84–1.16)	0.851
Hyperlipidaemia	2.3 (136)	2.7 (336)	2.70	0.100	0.84 (0.69–1.03)	0.091
Hypertension	5.9 (356)	6.3 (794)	1.12	0.290	0.93 (0.83–1.06)	0.276
Overweight, obesity, and other hyperlimentation	1.4 (86)	1.6 (202)	0.76	0.385	0.89 (0.69–1.14)	0.351
Any cardiometabolic disorder	12.3 (737)	12.8 (1607)	1.11	0.293	0.96 (0.88–1.04)	0.282
Females	*N* = 5723	*N* = 11,960				
Cerebrovascular disorders	2.0 (112)	1.9 (223)	0.13	0.717	1.05 (0.84–1.31)	0.673
Coronary artery disease	1.0 (60)	1.1 (136)	0.20	0.652	0.92 (0.68–1.25)	0.598
Diabetes mellitus	2.0 (115)	2.3 (285)	2.28	0.131	0.84 (0.68–1.04)	0.118
Hyperlipidaemia	0.9 (54)	1.1 (131)	0.72	0.396	0.86 (0.63–1.18)	0.353
Hypertension	5.0 (287)	4.9 (581)	0.17	0.678	1.03 (0.9–1.18)	0.651
Overweight, obesity, and other hyperlimentation	2.1 (119)	2.2 (265)	0.28	0.598	0.94 (0.76–1.16)	0.560
Any cardiometabolic disorder	9.8 (559)	9.7 (1157)	0.03	0.865	1.01 (0.92–1.11)	0.844

Cumulative incidences % (N), chi-square test (χ2) with p-value and risk ratio (RR) with 95% CI.

**Table 3.** Age of onset (Median (IQR)) and difference in medians (Quantile estimation Q) with a *p*-value of cardiometabolic disorders in NFBC1966 and the comparison cohort at age 7 to 50 by sex.

Diagnostic group	Males				Females			
	NFBC1966 *N* = 6000	Comparison cohort *N* = 12 511	Q	*p* Value	NFBC1966 *N* = 5723	Comparison cohort *N* = 11 960	Q	*p* Value
Cerebrovascular disorders	43.6 (36.1–46.7)	42.3 (36.3–46.4)	1.86	0.172	42.8 (37.8–47.5)	43.1 (36.3–47.1)	0.05	0.821
Coronary artery disease	44.4 (37.6–47.2)	44.9 (40.4–47.7)	0.35	0.549	42.5 (32.3–46.5)	45.8 (41.1–48.3)	3.85	0.049
Diabetes mellitus	42.9 (31.5–46.9)	42.9 (32.8–46.9)	0.01	0.928	38.1 (23.1–43.9)	41.7 (28.5–46.5)	8.28	0.004
Hyperlipidaemia	45.8 (42.4–48.0)	46.3 (43.0–46.7)	0.82	0.366	46.1 (43.9–48.0)	46.4 (43.4–48.5)	0.10	0.757
Hypertension	44.8 (40.6–47.4)	44.6 (41.4–48.0)	1.86	0.173	43.8 (38.4–46.9)	44.7 (39.5–47.7)	1.78	0.182
Overweight, obesity, and other hyperlimentation	44.3 (40.4–46.7)	44.2 (47.3–39.8)	0.01	0.938	43.8 (38.2–47.4)	44.2 (40.5–47.1)	0.10	0.780
Any cardiometabolic disorder	43.4 (36.6–46.8)	43.5 (37.4–45.2)	0.05	0.822	42.1 (34.4–46.4)	43.1 (35.5–47.2)	4.16	0.041

**Table 4.** Diabetes mellitus from age 22 to 50 years in NFBC1966 and comparison cohort by sex.

Diagnostic group	NFBC 1966		Comparison cohort					
	% (*N*)	Age of onset Median (IQR)	% (*N*)	Age of onset Median (IQR)	RR (95% CI)	*p* Value	Q	*p* Value
Males								
Type 1	1.3 (79)	31.7 (25.7–40.5)	1.4 (173)	33.3 (27.7–39.5)	0.95 (0.73–1.24)	0.716	2.35	0.125
Type 2	2.7 (163)	44.6 (40.3–47.4)	2.8 (355)	44.5 (40.1–47.6)	0.96 (0.80–1.15)	0.641	0.00	0.962
Unspecific Type	0.5 (30)	42.8 (39.0–46.5)	0.5 (59)	43.4 (36.9–47.0)	1.06 (0.68–1.64)	0.794	0.11	0.741
Females								
Type 1	0.9 (49)	29.1 (24.8–32.2)	0.9 (106)	31.5 (24.2–38.6)	0.97 (0.69–1.35)	0.841	3.73	0.053
Type 2	1.5 (85)	42.3 (37.5–45.9)	1.8 (220)	43.3 (37.9–47.2)	0.81 (0.63–1.04)	0.091	1.09	0.297
Unspecific Type	0.2 (10)	40.4 (28.5–43.1)	0.2 (29)	42.4 (37.9–46.8)	0.72 (0.35–1.48)	0.369	0.22	0.635

Cumulative incidences % (*N*), the median age of first onset (Median (IQR)), Risk Ratio (RR) with 95% CI and quantile estimation (Q) with the *p*-value.

**Figure 2.**
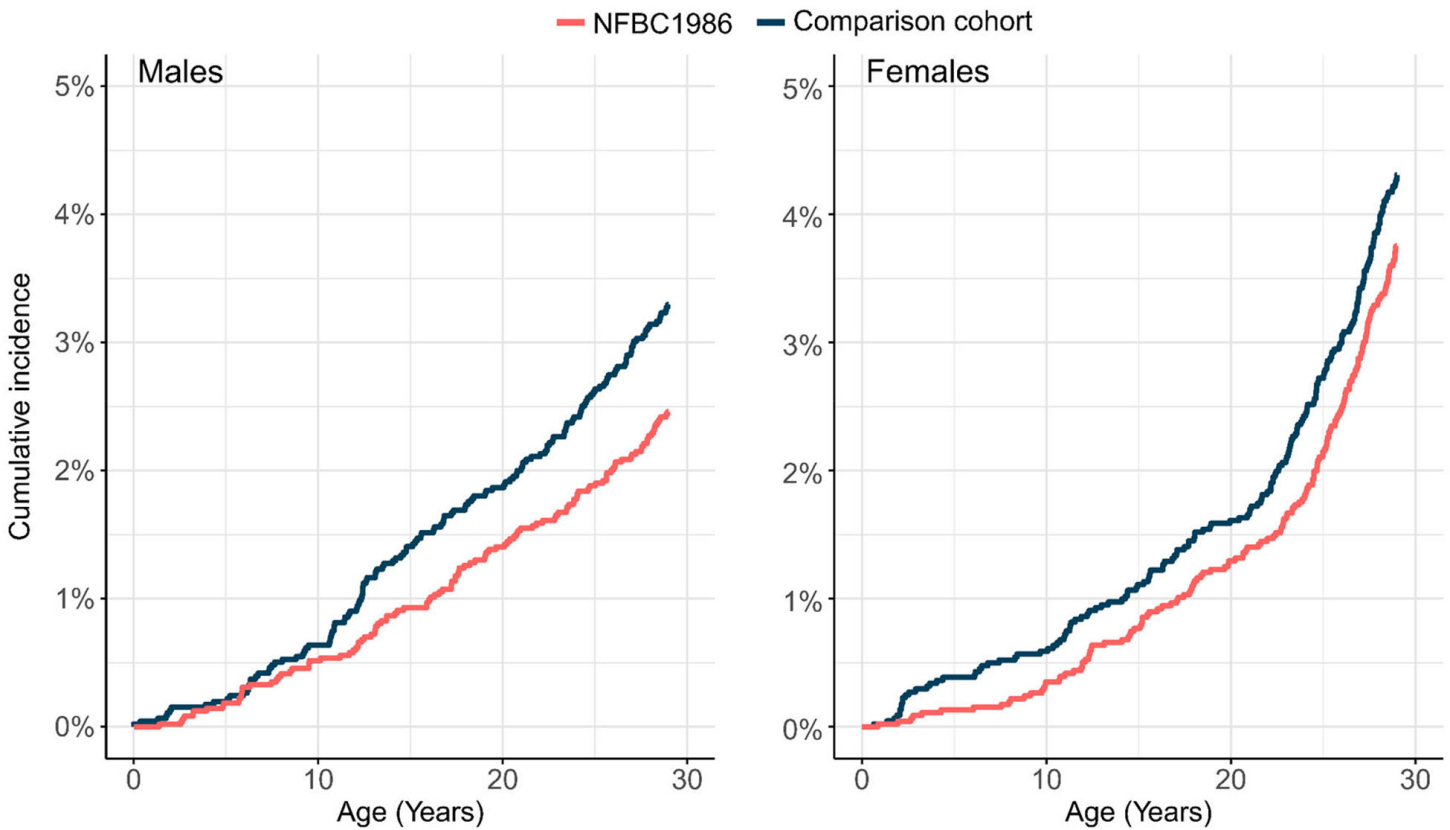
Cumulative incidence of first cardiometabolic disorders in NFBC1986 and comparison cohort at age of 0 to 29 years by sex.

The cumulative incidences of cardiovascular disorders (age 0 to 29 years) in NFBC1986 and its comparison cohort are reported in Table 4. Male members of NFBC1986 had a lower risk of overweight, obesity, and other hyperalimentation (RR: 0.45, 95% CI: 0.27–0.75), type 2 diabetes (RR 0.41, 95% CI: 0.27–0.75) and any cardiometabolic disorder (RR: 0.75, 95% CI: 0.59–0.95). In female members of NFBC1986, no difference in cumulative incidence rates between NFBC and the comparison cohort was found (Table 5). Figure 2 plots the age of the first onset of cardiometabolic disorders in the NFBC1986 and comparison cohort by sex. Male members of NFBC1986 had a higher age of onset in obesity (median age of onset 21.3 years vs. 15.1 years; Q: 10.76, *p* = 0.001) and any cardiometabolic disorder (median age of onset 17.6 years vs. 17.0 years; Q: 5.29, *p* = 0.022) and female members in diabetes mellitus (median age of onset 15.1 years vs.10.1 years; Q: 8.06, *p* = 0.005) than the comparison cohort. (Table 6).

**Table 5.** Cardiovascular disorders in NFBC1986 with a comparison cohort at age 0 to 29 years.

Diagnostic group	NFBC 1986	Comparison cohort	*χ^2^*	*p* Value	RR (95% CI.)	*p* Value
Males	*N* = 4839	*N* = 4550				
Cerebrovascular disorders	0.4 (20)	0.4 (18)	0.00	0.999	1.04 (0.55–1.97)	0.893
Diabetes mellitus	1.0 (50)	1.5 (66)	3.01	0.083	0.71 (0.49–1.03)	0.067
Type 1^a^	0.9 (46)	1.2 (53)	0.84	0.360	0.82 (0.55–1.21)	0.315
Type 2^a^	0.2 (10)	0.5 (23)	5.16	0.023	0.41 (0.20–0.86)	0.015
Unspecific type^a^	0.1 (5)	0.1 (4)				
Hypertension	0.5 (24)	0.7 (30)	0.83	0.363	0.75 (0.44–1.28)	0.295
Overweight, obesity, and other hyperalimentation	0.5 (22)	1.0 (46)	9.34	0.002	0.45 (0.27–0.75)	0.001
Any cardiometabolic disorder	2.5 (119)	3.3 (150)	5.61	0.018	0.75 (0.59–0.95)	0.015
Females	*N* = 4557	*N* = 4409				
Cerebrovascular disorders	0.3 (13)	0.2 (10)	0.11	0.735	1.26 (0.55–2.87)	0.584
Diabetes mellitus	0.9 (43)	1.1 (47)	0.23	0.635	0.89 (0.59–1.34)	0.561
Type 1^a^	0.8 (36)	0.8 (37)	0.02	0.887	0.80 (0.60–1.49)	0.800
Type 2^a^	0.2 (11)	0.3 (14)	0.23	0.629	0.76 (0.35–1.67)	0.494
Unspecific type^a^	0.0 (2)	0.2 (9)				
Hypertension	0.4 (16)	0.5 (20)	0.36	0.548	0.77 (0.4–1.49)	0.443
Overweight, obesity, and other hyperalimentation	2.1 (96)	2.6 (115)	2.24	0.134	0.81 (0.62–1.06)	0.117
Any cardiometabolic disorder	3.8 (171)	4.3 (190)	1.66	0.198	0.87 (0.71–1.07)	0.180

Cumulative incidences % (*N*), Pearson’s chi-square statistics (χ^2^), and Risk Ratio (RR) with 95% CI.

^a^At age 2 to 29 years.

**Table 6.** Age of onset (Median (IQR)) and difference in medians (Quantile estimation Q) with a *p*-value of cardiometabolic disorders in NFBC1986 and comparison cohort at age 0 to 29 by sex.

Diagnostic group	Males				Females			
	NFBC1986 *N* = 4839	Comparison cohort *N* = 4550	Q	*p* Value	NFBC1986 *N* = 4557	Comparison cohort *N* = 4409	Q	*p* Value
Cerebrovascular disorders	20.9 (11.2–26.7)	24.7 (21.4–26.4)	1.94	0.163	20.9 (22.3–25.7)	20.7 (17.6–22.0)	0.00	0.976
Diabetes mellitus	14.5 (7.5–20.2)	13.9 (8.9–21.5)	0.14	0.704	15.1 (9.9–21.8)	10.1 (3.6–19.7)	8.06	0.005
Type 1^a^	12.9 (6.6–17.6)	12.2 (8.7–18.2)	0.21	0.654	12.0 (9.53–19.9)	8.3 (3.21–13.54)	3.65	0.056
Type 2^a^	22.4 (20.8–23.8)	23.8 (20.9–26.7)	0.40	0.529	23.8 (22.2–26.1)	21.4 (17.5–24.9)	0.56	0.452
Hypertension	23.6 (19.1–25.6)	23.0 (14.6–24.8)	0.17	0.684	24.9 (22.1–26.1)	23.1 (20.4–25.8)	1.22	0.269
Overweight, obesity, and other hyperalimentation	21.3 (13.1–26.1)	15.1 (12.2–25.8)	10.76	0.001	26.1 (23.9–27.5)	25.0 (22.5–27.3)	2.21	0.137
Any cardiometabolic disorder	17.6 (12.1–23.4)	17.0 (11.4–24.2)	5.29	0.022	24.2 (16.5–26.8)	23.2 (14.9–26.7)	1.95	0.163

^a^At age 2 to 29 years.

Cardiometabolic-related deaths in the NFBC1966 and comparison cohort by sex are reported in Table 7. No significant difference between NFBC1966 and the comparison cohort was found (males RR: 1.06, 95% CI: 0.68–1.64, *p* = 0.794; females RR: 1.93, 95% CI: 0.88–4.22, *p* = 0.095). Age of death did not differ between NFBC1966 and the comparison cohort in males (Q: 0.50, *p* = 0.481) or females (Q: 0.00, *p* = 0.973).

**Table 7.** Cumulative incidence % (*N*) of cardiometabolic-related deaths, and age of death caused by any cardiometabolic disorder at age 0 to 50 years in NFBC1966 with comparison cohort by sex.

	Males		Females	
Cause of death	NFBC1966 *N* = 6000 % (*N*)	Comparison *N* = 12,511 % (*N*)	NFBC1966 *N* = 5723 % (*N*)	Comparison *N* = 11,960 % (*N*)
Cerebrovascular disorders	0.1 (6)	0.1 (10)	0.1 (4)	0.1 (6)
Coronary artery disease	0.3 (18)	0.3 (41)	0.1 (4)	0.0 (4)
Diabetes mellitus	0.1 (4)	0.0 (2)	0.1 (4)	0.0 (2)
Hypertension	0.0 (2)	0.0 (6)	0.0 (0)	0.0 (1)
Death caused by any cardiometabolic disorder	0.5 (30)	0.5 (59)	0.2 (12)	0.1 (13)
Age of death (Median (IQR))	42.6 (36.1–46.7)	44.1 (38.2–47.9)	43.0 (41.6–46.2)	42.9 (40.0–44.3)

## 4. Discussion

The results partly supported our hypothesis of lower incidences of cardiometabolic disorders in the NFBC, where the male members of NFBC1986 had decreased risk of overweight, obesity, and other hyperalimentation, type II diabetes and any cardiometabolic disorder diagnosis at age of 0 to 29 years. In the NFBC1966 study, there was no significant difference in the overall incidence of coronary artery disease, diabetes mellitus, or any cardiometabolic disorder between the study and comparison cohorts. However, the onset of these disorders occurred at a younger age in females within the study cohort. This may indicate that female members of NFBC1966 tended to seek medical treatment more frequently, potentially due to the follow-up studies conducted in NFBC1966. The NFBC1966 participants had more frequent measurements of cardiometabolic risk factors during the follow-up (clinical examinations at ages 31 and 46 years), which could have contributed to the earlier age of onset of cardiometabolic disorders in NFBC1966. It is also possible that members of NFBC1966 are generally more self-aware of their health, leading to earlier detection and treatment of these conditions. Further research is needed to fully understand the factors that may have contributed to the observed differences in age of onset between the study and comparison cohorts.

The follow-ups of the NFBC studies differ considerably. In NFBC1986, the follow-up was more intense in childhood and adolescence (follow-ups for the whole cohort at age of 7, 8, and 15–16 years), while in NFBC1966 the follow-ups for the whole cohort included childhood and adolescent data collection only at age 14 with questionnaire years. The age 15–16 years follow-up study of NFBC1986 explored possible cardiovascular risk groups by surveying health, lifestyle choices, and eating habits widely. The follow-up study also included a clinical examination, in which blood pressure, height, and weight were measured and a physical fitness test was conducted. In NFBC1966, the follow-up at age of 14 years included only basic questions about activity, BMI, and substance use. The data collection regarding cardiovascular risk factors was limited. Moreover, the first clinical examination in the follow-up for the whole cohort in NFBC1966 was conducted during the age 31 years follow-up study. The younger cohort might have been influenced by information on healthier lifestyle choices in adolescence *via* follow-ups conducted at age 15–16 years. In the age 31 and 46 years follow-ups of NFBC1966, eating habits, physical exercise, and overall health were explored widely, but there was no difference in cardiometabolic disorders from age 7 to 50 years between NFBC1966 and the comparison cohort.

Eurostat reports that the prevalence of self-reported overweight and obesity (BMI ≥ 25) was 39.3% among Finnish males aged 18 to 29 years and 66.0% among Finnish males aged 25 to 64 years in 2014 [28]. Obesity is a relatively rare cause to use healthcare: 0.5% in NFBC1986 and 1.0% in the comparison cohort had a healthcare diagnosis of overweight, obesity, and other hyperalimentation until the age of 29; 1.3% in males in NFBC1966, 1.5% in the comparison cohort from age of 7 to 50 years. Diagnoses related to overweight and obesity cases are seldom given in specialised care. Nevertheless, obesity is preventable *via* lifestyle choices. The systematic review suggests that population reductions in weight are achievable through community-based interventions, including interventions that have incorporated educational, health promotion, social marketing, policy, or legislative reform strategies [29]. Multiple follow-up studies conducted on NFBC1986 during childhood and adolescence might have encouraged the participants of NFBC1986 to make healthier lifestyle choices, and therefore, the male members of NFBC1986 did have less overweight, obesity, or another hyperalimentation diagnosis, and were diagnosed at an older age than the comparison cohort.

Type 1 and type 2 diabetes develop due to interactions between genetic and environmental factors, but more behavioural factors, such as a sedentary lifestyle and poor diet, have been associated with type 2 diabetes [30,31]. Type 1 diabetes occurs predominantly in young people (diagnosis at 30 years of age or younger) [32], whereas type 2 diabetes generally manifests after age 40 years [33]. Male members of NFBC1986 had a lower risk for type 2 diabetes and female members had a higher age of onset of diabetes mellitus than the comparison cohort, but that was mostly due to the age at type 1 diabetes mellitus diagnosis. If the follow-up has encouraged the participants to make healthier lifestyle choices, it has likely affected the onset of type 2 diabetes, rather than type 1 diabetes.

In NFBC1966, no clear peaks were seen in the cumulative incidences of cardiometabolic disorders during the follow-up period. In NFBC1986, the difference in the cumulative incidence of cardiometabolic disorders between the study cohort and the comparison cohort appeared to steadily increase in males over the follow-up period, having a higher incidence in the comparison cohort. In females, in the comparison cohort cumulative incidence of cardiometabolic disorders peaked at an early age and then the difference between NFBC1986 and the comparison cohort remained stable over time. However, the members of NFBC1986 were a little too young for an accurate assessment of the differences in the incidence of cardiometabolic disorders. The preliminary results for NFBC1986 suggest possible health-promoting effects, but these need to be confirmed as the cohort members age.

### 4.1. Strengths

The data used in this study were from Finnish registers, which have been found to have a good standard [26]. The NFBC studies and comparison cohorts were born in the same area one to two years apart, so it could be assumed that the sociodemographic background factors did not vary across the cohorts. The response rate in the NFBC studies can be considered high since poor response rates in follow-up cohort studies are causing increasing concern [34]. In addition, the study included two large birth cohorts, in which the follow-ups differ from each other.

### 4.2. Limitations

The study also has some limitations. We could not identify outpatient and inpatient hospital visits in NFBC1966 and its comparison cohort from 1998 onwards. The comparison cohort of NFBC1966 includes a small percentage (1.0%) of original members of NFBC1966. The prevalence rates were also relatively low in some diagnostics groups, especially for the younger birth cohort, which could have led to false-positive findings.

## 5. Conclusion

The results partly supported our hypothesis of lower incidences of cardiometabolic disorders in NFBC, where the male members of NFBC1986 had decreased risk of overweight, obesity, and other hyperalimentation, type 2 diabetes and any cardiometabolic disorder diagnosis at age of 0 to 29 years. However, the members of NFBC1986 were a little too young for an accurate assessment of the differences in the incidence of cardiometabolic disorders. The preliminary results for NFBC1986 suggest possible health-promoting effects, but these need to be confirmed as the cohort members age. The participation bias in epidemiological follow-up studies has previously been under-examined, and the results need to be replicated. In a previous study, an association between the use of mental health care services and participation in the NFBC1986 study was found [35].

## Supplementary Material

Supplemental Material

## Data Availability

Data cannot be shared publicly because the utilised data have been given for this specific study, and the data cannot be shared without permission from the University of Oulu and Findata. The register data can be applied from Findata, the Finnish Health and Social Data Permit Authority by researchers who meet the criteria for access to confidential data. NFBC data is available from the University of Oulu, Infrastructure for Population Studies. Permission to use the data can be applied for research purposes *via* the electronic material request portal. In the use of data, we follow the EU general data protection regulation (679/2016) and Finnish Data Protection Act. The use of personal data is based on cohort participants written informed consent in his/her latest follow-up study, which may cause limitations to its use. Please, contact the NFBC project centre (NFBCprojectcenter(at)oulu.fi) and visit the cohort website for more information. Aggregated data underlying the results presented in the study can be asked from the corresponding author as long as the current permission is valid (2025).
