# Supplementary material for: Association between participation in the Northern Finland Birth Cohorts and cardiometabolic disorders
Source: Ann Med. 2023 Mar 22;55(1):1123–33. doi: 10.1080/07853890.2023.2186478 (PMC10035958; doi:10.1080/07853890.2023.2186478)
Supplement: Supplemental Material [file IANN_A_2186478_SM6782.docx]

**SUPPLEMENT MATERIAL**

Supplement table 1 Questions of the follow-up at the age of 14-years in the NFBC1966 regarding physical exercise, diet, cardiovascular health, and substance use

| Question | Answer options |
| --- | --- |
| **Physical exercise** | |
| How often are you interested in one or more sports outside school? | Every day; Every second day; Twice a week; Once a week; Every second week; Once a month; Usually never |
| What is the type of sports you mainly do? |  |
| Are you a member of a sport club? | Yes; No |
| Did you ever win a prize in sports event? | No; Once or twice; Three or more times |
| The mark you had in your report card for the spring term 1980 in gymnastics and sport |  |
| **Health** | |
| Your weight and height? |  |
| **Substance use** | |
| Smoking | I have never tried; I tried once; I have tried twice or more; I smoke occasionally; I smoke about twice a week; I smoke 1-5 cigarettes daily; I smoke 6-10 cigarettes daily; I smoke more than 10 cigarettes daily |
| Alcohol use (beer or other alcohol beverages) | I have never drunk any; I tasted once; I have drunk few times; I use alcohol monthly; I use alcohol weekly |
| Other intoxicants (e.g., thinner or drugs) | I have not tried any; I have tried some; I have used several times; I use regularly |

Supplement table 2 Questions of the follow-up at the age of 31-years in the NFBC1966 regarding physical exercise, diet, cardiovascular health, and substance use

| Question | Answer options |
| --- | --- |
| **Physical exercise** | |
| How well are you capable of doing the following things? Running 5km without a rest; Running 2 km without a rest; Lifting and carrying heavy weights (women; more than 17kg, men; more than 25kg); Squatting and standing up 20 times; Bending forward while standing knees straight – fingers touching the ground; Lying on your back and getting into a sitting position with your legs straight without using your hands? | No problems; I can but it is difficult; I can, but it is very difficult; I can’t at all |
| How often in your leisure time do take exercise? Light exercise; Keep-fit exercise | Once a month or less; 2-3 times per month; Once a week; 2-3 times per week; 4-6 times per week; Daily |
| How long at a time do you take exercise in your leisure time? Light exercise; Keep-fir exercise | Never; Less than 20 minutes; 20-39 minutes; 40-59 minutes; 1-1.5 hours; more than 1.5 hours |
| **Diet** | |
| Where do you mostly eat your lunch? | I don’t have lunch; at home; I eat packed lunch at work; at my workplace canteen; in a restaurant fast-food restaurant; elsewhere, where? |
| Do you usually put milk or cream in your coffee and/or tea? | Neither; milk; cream |
| How many glasses do you usually drink or eat in a day? milk; sour milk; other milk products |  |
| How many slices of cheese do you eat in a day? |  |
| How often do you eat the following foods? Grain products (6 items); Milk products (6 items); Vegetables (5 items); Fish, meat, eggs (7 items); Fruits, berries (2 items); Others (6 items) | Less than once a month or never; Once or twice a month; once a week; a couple of times a week; Nearly every day; Once a day or more often |
| **Health** | |
| Your weight and height? |  |
| Your own estimate about your health right now? | Very good; Good; Moderate; Bad; Very bad |
| Have you ever had any of the following symptoms, sicknesses or injuries verified or treated by a doctor? 34 items, e.g.: Elevated blood pressure, hypertension; Congenital heart disease; Cardiac insufficiency; Chest pain up on strain; Diabetes; Thyroiditis | Yes; No |
| To what extent does you sickness or symptoms disturb your daily life? | Not at all; Quite a little; A little; Quite a lot; Very much |
| Hof often do you use the following medication? 11 items, e.g.: Medication for cardiac and circulatory complaints | Not at all; Sometimes; Regularly or continually |
| **Substance use** | |
| Do you drink even occasionally alcoholic beverages? | I have never used alcohol; no because I have up alcohol completely xx years ago; Yes, less often than once a month; Yes, at least once a month |
| How often do you usually drink beer, cider or long drinks; light wine; wine; spirits? | Never; Once a year or less often; A couple of times a year; 3-4 times a year; Once in a couple of months; Once a month; A couple times of month; Once a week; A few times a week; Daily |
| How much do you usually drink beer, cider or long drinks; light wine; wine; spirits? |  |
| Have you ever smoked in your life? | No; Yes, I started when I was xx years old |
| Have you ever been smoking regularly? | No; Yes, I have smoked regularly for altogether xx years |
| If you have given up smoking, how old were you when you quit? |  |
| Do you smoke nowadays? | On 7 days a week; On 5-6 days a week; On 2-4 days a week; On one day a week; Occasionally; Not at all |
| When did you last smoke? | Yesterday or today; 2 days – 1 month ago; 7-11 months ago; 1-5 years ago; 6-10 years ago; More than 10 years ago |
| How much per day do you usually smoke now or smoked before you gave up smoking? filter cigarettes; other cigarettes; pipefuls; cigars |  |
| Do you use snuff od chewing tobacco? | Yes, regularly; Occasionally; Not at all |
| How many hours a day do you spend in premises where you have to inhale smoke produced by other people smoking? |  |

Supplement table 3 Questions of the follow-up at the age of 46-years in the NFBC1966 regarding physical exercise, diet, cardiovascular health, and substance use

| Question | Answer options | |
| --- | --- | --- |
| **Physical exercise** | | |
| How often in your leisure time do take exercise? Light exercise; Keep-fit exercise | | Once a month or less; 2-3 times per month; Once a week; 2-3 times per week; 4-6 times per week; Daily |
| How long at a time do you take exercise in your leisure time? Light exercise; Keep-fir exercise | | Never; Less than 20 minutes; 20-39 minutes; 40-59 minutes; 1-1.5 hours; more than 1.5 hours |
| How much do you exercise and strain yourself physically in your leisure time? | | In my leisure time, I read, watch television and do chores that do not involve much movement or straining myself physically; In my leisure time, I walk, ride a bike or do other types of exercise at least for four hours a week; In my leisure time, I do actual fitness training, such as running, jogging, skiing, gymnastics, swimming an ball games on average at least for two hours a week; In my leisure time, I regularly do competitive training several times a week. |
| How many hours do you sit on average during weekdays? During the workday at the office such place; At home watching TV or videos; At home at the computer; In a vehicle; Other | |  |
| How well are you capable of doing the following things? Running 5km without a rest; Running 2 km without a rest; Lifting and carrying heavy weights (women; more than 17kg, men; more than 25kg); Squatting and standing up 20 times; Bending forward while standing knees straight – fingers touching the ground; Lying on your back and getting into a sitting position with your legs straight without using your hands? | | No problems; I can but it is difficult; I can, but it is very difficult; I can’t at all |
| How often do you engage in the following types of sports and exercise? Fitness training (16 items); Other activities involving physical strain (5 items) | | Not at all; Once a month or less; 2-3 times a month; Once a week; 2-3 times a week; 4 times a week or more |
| **Diet** | | |
| Do you normally eat the following meals? Breakfast; Lunch; Dinner; Light snack on the evening; Snacks between meals; Night-time meal; Snacks during the night | | On weekdays: No; Yes, On weekends: No; Yes |
| How many snacks do you eat in a day? On weekdays; On weekends; On the night sift | |  |
| Where do you most often eat your lunch? | | I don’t have lunch; at home; I eat packed lunch at work; at my workplace canteen; in a restaurant; elsewhere, where? |
| Do you have a special diet? Lactose-free; Gluten-free; Food allergy; Diabetic diet; Cholesterol-lowering diet; Weight-loss diet; Vegetarian; Low-sodium diet; Other | | No; Yes |
| How often do you eat the following foods? Grain products (6 items); Dairy products (6 items); Vegetables (5 items); Fish, meat, eggs (9 items); Fruits, berries (2 items); Others (9 items) | | Less than once a month or never; Once or twice a month; once a week; a couple of times a week; Nearly every day; Once a day or more often |
| How often do you avoid having tempting food around? | | Almost never; Rarely; Often; Almost all of the time |
| How likely is that you could consciously eat less than you want to? | | Unlikely; Not very likely; Somewhat likely; Very likely |
| Do you keep eating even though you are not hungry? | | Never; Rarely; Sometimes; At least once a week |
| On a scale 1 (I eat what I want whenever I want) to 8 (I constantly limit my eating) which number are you? | |  |
| How much fat do you put on a slice of bread? (Refer to the picture for help) | | Not at all; amount xx |
| Which butter/margarine is normally used in your home in cooking? (not including baking)? | |  |
| If you drink milk, which type of milk do you use? | |  |
| If you drink sour milk, which type of sour milk do you use? | |  |
| How many glasses of milk products do you normally drink or eat per day? milk; sour milk; other milk products | |  |
| How many slices of low fat (17% or less) cheese do you normally eat per day? | |  |
| How many slices of normal-fat (more than 17%) cheese do you normally eat per day? | |  |
| How many slices of bread do you normally eat per day? Rye bread; Mixed grain, yeast, graham, barley, or oat bread, whole wheat baguette; French bread, wheat toast, white-flour baguette | |  |
| If you eat soured whole milk or yoghurt, which type do you use? | |  |
| In the last 12 months, have you drunk any raw cow’s milk (farm milk)? | | No; Yes, daily; Yes, occasionally |
| Did you drink raw cow’s milk when you were under seven years of age? | | No; Yes, daily; Yes, occasionally |
| **Health** | | |
| Your weight and height? | |  |
| Your own estimate about your health right now? | | Very good; Good; Moderate; Bad; Very bad |
| Have you ever had any of the following symptoms, sicknesses or injuries verified or treated by a doctor? Cardiovascular diseases (High blood pressure, hypertension; Congenital heart disease; Congestive heart failure; Coronary artery disease); Diabetes (Type 1; Type 2); Thyroid dysfunction (2 items); Gastric and intestinal diseases (3 items); Skin diseases (3 items); Infections (8 items); Ear disease or trauma to the ear; Eye diseases and symptoms (10 items); Brain and nervous system diseases (4 items); Cancers (2 items); Hernia; Anemia; Oral diseases and disorders (2 items); Mental health (3 items); Substance abuse (2 items); Sleep apnea; Musculoskeletal disorders and rheumatic diseases (25 items) | | Yes; No |
| Have you experienced any pain in your chest that occurs under strain within the last 12 months? | | No; Yes |
| Hof often do you use the following medication? 11 items, e.g.: Medication for cardiac and circulatory complaints | | Not at all; Sometimes; Regularly or continually |
| Have you ever seriously tried to lose weight? | | Never; Yes, once; Yes, several times |
| If you have lost weight, when was the last time? | | More than a year ago; Six months to a year ago; One month to six months ago; Within the last month |
| How do you feel about your weight? | | Significantly overweight; Slightly overweight; Just the right weight; Slightly or significantly underweight |
| Describe your state of health today: Mobility | | I can walk normally indoors, outdoors and on the stairs; I can walk without difficulty, but have minor difficulties walking outdoors and/or the stairs; I van walk indoors without assistance, but have considerable difficulties or need assistance when walking outdoors and/or in the stairs; Even indoors, I am only able to walk if I am assisted by someone; I am totally unable to move and have to stay in bed |
| **Substance use** | | |
| Do you drink even occasionally alcoholic beverages? | | I have never used alcohol; no because I have up alcohol completely xx years ago; Yes, less often than once a month; Yes, at least once a month |
| How often do you usually drink beer, cider or long drinks; light wine; wine; spirits? | | Never; Once a year or less often; A couple of times a year; 3-4 times a year; Once in a couple of months; Once a month; A couple times of month; Once a week; A few times a week; Daily |
| How much do you usually drink beer, cider or long drinks; light wine; wine; spirits? | |  |
| Have you ever smoked in your life? | | No; Yes, I started when I was __ years old |
| Have you ever been smoking regularly? | | No; Yes, I have smoked regularly for altogether xx years |
| If you have given up smoking, how old were you when you quit? | |  |
| Do you smoke nowadays? | | On 7 days a week; On 5-6 days a week; On 2-4 days a week; On one day a week; Occasionally; Not at all |
| When did you last smoke? | | Yesterday or today; 2 days – 1 month ago; 7-11 months ago; 1-5 years ago; 6-10 years ago; More than 10 years ago |
| How much per day do you usually smoke now or smoked before you gave up smoking? filter cigarettes; other cigarettes; pipefuls; cigars | |  |
| Do you use snuff od chewing tobacco? | | Yes, regularly; Occasionally; Not at all |
| How many hours a day do you spend in premises where you have to inhale smoke produced by other people smoking? | |  |

Supplement table 4 Sub-studies of the Northern Finland Birth Cohort 1966 (NFBC1966).

| Age (main reference) | Target Population  *n* | Questionnaire data  (Participation rate) | Clinical Examination  (Participation rate) |
| --- | --- | --- | --- |
| 1 year ^12^ | Children with perinatal risk *n*=793 | N/A | Neurological examination *n*=722 (91%) |
| 14 years ^36^ | Children with low school performance *n*=495 | N/A | IQ-test |
| 16 years ^37^ | Unwanted children (*n*=231) with control group (*n*=227) | Questionnaire to unwanted children’s *n*=88 (38.1%) and controls *n*=89 (39.2%) teachers | N/A |
| 15 years ^38^ | Children with known myopia *n*=707 and their controls *n*=784 | N/A | Ophthalmological examination to cases *n*=236 (33.4%) and controls *n*=266 (33.9%) |
| 19 years ^39^ | Twins and their controls *n*=652 | Questionnaire at age of 19 *n=652* | N/A |
| 24 years ^16^ | Random subsample of males *n*=2500 | On health and life satisfaction *n*=2500 | N/A |
| 31 years ^40^ | Living in the city of Oulu *n*=1609 | SCID II | SCID I and SCID II interviews |
| 31 years ^17^ | Participated in 31-year clinical examination in the city of Oulu *n*=1609 | N/A | Bone mineral density and content measurements *n=*1102 (68.5%) |
| 31 years ^17^ | Random sample based on 31-years questionnaire *n*=196 | Exercise and food diaries for 7 days | N/A |
| 31 years^a^ | Subjects with problems in hearing at age of 14 and their controls *n*=1372 | N/A | Audiogram studies |
| 33 years ^41^ | Subjects with psychosis and their controls *n*=191 | Psychiatric interview and questionnaire | Brain MRI scans and cognitive test |
| 39 years^a^ | All men alive | A postal questionnaire regarding loss of hair (*n*=3128) | N/A |
| 42 year ^41^ | Subjects with psychosis, their controls and siblings *n*=312 | Psychiatric interview and questionnaire | Brain magnetic resonance imaging (MRI) scans and cognitive test |
| 48 year ^42^ | Random sample of the cohort was invited to the eye screening *n*=5155 | N/A | Eye screenings, *n=*3070 |
| ^a^ Data have not been published | | | |

Supplement table 5 Questions of the follow-up at the age of 15-16 in the NFBC1986 regarding physical exercise, diet, cardiovascular health, and substance use

| Question | Answer options |
| --- | --- |
| **Physical exercise** | |
| Outside the school hours, how many hours a day do you spend at the following activities (hours/day)? | Watching television; Reading books or papers; Playing computer or video games/working with computer; Other things that you do mostly sitting |
| How many hours do you usually sleep per day (24 hours)? |  |
| Do you belong to a sport club? | No; Yes, but I do not attend the training sessions; Yes, and I attend the training sessions |
| How many times altogether does it take you to walk, bike or otherwise physically move to get to school and home from school daily? | Not at all; Less than 20 minutes a day; 20 – 39 minutes a day; 40 -59 minutes a day; more than an hour day |
| Outside the school hours, how often in your free time do you do strenuous physical exercise for at least 20 minutes at time? | Never; Once a month or less often; 2-3 times a month; Once a week; Twice a week; 3 times a week; 4-6 times a week; Daily |
| Outside the school hours an the time spend going to school and coming home from school, how many hours a week altogether do you spend in strenuous physical activity? | Not at all; About ½ hours a week; About an hour a week; 2-3 hours a week; About 4-6 hours a week; 7 hours a week or more |
| In addition to exercise described above, how many hours altogether do you spend doing light physical exercise? | Not at all; About ½ hours a week; About an hour a week; 2-3 hours a week; About 4-6 hours a week; 7 hours a week or more |
| How often do you generally do the following types of physical exercise outside the school hours? (27 different sport types) | Never; Once a month or less often; 2-3 times a month; Once a week; 2-3 times a week; 4 times a week or more often |
| **Diet** | |
| Do you usually have a following meals? Breakfast; Lunch (midday meal at 11 – 13); Dinner (late afternoon meal); Evening snack; Snacks between meals | On weekdays: No; Yes, During weekends: No; Yes |
| How many cups of coffee and/or tea do you drink in a day? |  |
| How many lumps of sugar or teaspoonfuls or granulated sugar do you put in cup of coffee and / or tea? |  |
| How many glasses of the following products do you drink or eat daily? Milk; Sour milk; Other milk products |  |
| How many slices of bread do you usually eat in a day? Dark bread; Brown, full-wheat or oatmeal bread; White bread |  |
| How many slices of cheese do you eat daily? |  |
| How often did you eat uncooked vegetables whole or grated or as a salad during the past week? | Not at all; On 1-2 days; On 3-5 days; On 6-7 days |
| How often did you eat uncooked fruit or fruit salad during the past week? | Not at all; On 1-2 days; On 3-5 days; On 6-7 days |
| How often did you eat berries during the past week? | Not at all; On 1-2 days; On 3-5 days; On 6-7 days |
| How often do you usually eat the following foodstuffs? Think back for the last six months (27 different items, including sweets, meats, soft drinks etc.) | Less often than once a month or never; Once or twice a month; Once a week ; 3-5 times a week; Almost daily; Once a day or more often |
| If you drink milk, which of the following do you usually drink? | I do not drink milk; Unprocessed milk from a dairy farm; Full milk from a carton; Low-fat milk; Milk with 1% fat; Non-fat milk |
| If you drink sour milk, which of the following do you drink? | I do not drink sour milk; Non-fat sour milk, butter milk; Asidofilus or Neo sour milk |
| What kind of spread do you mostly put on your bread? | Light spread with 40% or 60% fat; Vegetable stanole product; Margarine or spread with 70% or 80% fat; Baking margarine; Mixture of butter and vegetable oil; Oil; Butter; Nothing |
| How much fat do you put on a slice of bread? (Refer to the picture for help) | 2.5g; 5g; 10g; 15g; No fat |
| Are there foodstuffs that you cannot eat for some reason? | No; Yes, what and why? |
| Do you ever devour large amounts of food? | No; Yes |
| How often do you devour large amounts of food? | I never devour food; Hardly ever; Occasionally; Once a month; Once a week; 2-3 times a week; Daily |
| Do you feel miserable after having a devoured large amount of food? | I never devour food; I do not feel miserable afterwards; Yes, I do feel miserable afterwards |
| If you devour large amounts of food, do you do it alone? | I never devour food; Yes; No |
| Are there times in your life when you cannot think about anything except food? | No; Yes |
| When you feel stress about a difficult thing, event or situation, do you try to make yourself feel better by eating? | Never; Sometimes; Quite often; Very often |
| **Health** | |
| How would you describe your health at the moment? | Very poor; poor; Moderate; Good; Very Good |
| Have you ever been diagnosed for a congenital or other heart defect? | No; Yes, what kind? |
| Has a doctor or nurse found you have a high blood pressure? | No; Yes, when and where? |
| Your weight and height? |  |
| What is your opinion of your weight? Do you consider yourself | Clearly underweight; Slightly underweight; Normal; Slightly overweight; Notably overweight |
| Are you afraid of the idea of getting fat? | No; Yes |
| Do you use some of the following ways to control your weight? Strict diet/fasting; Heavy exercise; Vomiting; Laxatives or other medicines/slimming methods | Never; Occasionally; Often |
| **Substance use** | |
| Have you ever smoked or used snuff in your life? | No; Yes, but I have only tried; Yes I started smoking when I was xx years old; Yes, I started using snuff when I was xx years old |
| Have you ever smoked or using snuff regularly? | No; Yes, I have smoked regularly for xx years; Yes, I have used snuff regularly for xx years |
| Do you smoke now? | Not at all; Occasionally; On one day a week; On 2-4 days a week; On 5-6 days a week; On 7 days a week |
| Do you use snuff now? | Not at all; Occasionally; On one day a week; On 2-4 days a week; On 5-6 days a week; On 7 days a week |
| How much do you smoke or use snuff now? | Filter cigarettes xx a day; Other cigarettes xx a day; Snuff/quid xx times a day |
| How many hours do you spend daily in a room where you have to breathe in tobacco smoke from other smokers? |  |
| Have you ever drink or do you still drink even occasionally some alcoholic beverages? | I have never drunk alcohol; I have tasted alcohol, but do not drink now; I use alcohol occasionally; I use alcohol about once a month; I use alcohol 2-3 times a month; I use alcohol once a week or more often |
| How many times have you has at least one drink of alcohol? In your life; During the past 12 months; During the past 30 days | Never; 1-2 times; 3-5 times; 6-9 times; 10-19 times; 20-39 times; 40 times or more |
| How many times have you been drunk? In your life; During the past 12 months; During the past 30 days | Never; 1-2 times; 3-5 times; 6-9 times; 10-19 times; 20-39 times; 40 times or more |
| Use this scale to evaluate how drunk you were when you last were drunk | Only a little – So drunk I could hardly stand |
| How many drinks do you need to get drunk? | I have never drunk alcohol; I have never been drunk; 1-2 drinks; 3 drinks; 4 drinks; 5 drinks; 6 drinks; 7-8 drinks; 9 drinks or more |
| Think back for the past 30 days. How many times during that time have you drink six drinks or more on the same occasion? | Never; Once; Twice; 3-5 times; 6-9 times; 10 times or more |
| How often have you drunk beer, cider or long drinks; light wine; wine; spirits during past 12 months? | Never; A couple times a year; 3-4 times a year; About once in a couple of months; About once a month; A couple times a month; Once a week; 2-3 times a week; 4-5 times a week; 6-7 times a week |
| How much did you usually drink beer, cider or long drinks; light wine; wine; spirits? |  |
| Have you ever been offered drugs? | Never; Once; Several times |
| Have you ever tried or used any of the following substances? Medicines for intoxication; Alcohol and pills together; Sniffing for intoxication; Marijuana or hashish; Ecstasy, heroin, cocaine, amphetamine, LSD or other similar drugs; Intravenously injected drugs? | Never; Once; 2-4 times; 5 times or more; I use regularly |
| At what age did you do the following things for the first time? Drank beer; Drank wine; Drank spirits; Got drunk; Smoked your first cigarette; Began to smoke daily; Tried an intoxicating substance (thinner, glue, hashish, etc.) | Never; 11 years or under; 12 years; 13 years; 14 years; 15 years; 16 years |

Supplement table 6 Sub-studies of the Northern Finland Birth Cohort 1986 (NFBC1986).

| Age (main reference) | Target Population  N | Questionnaire data  (Participation rate) | Clinical Examination  (Participation rate) |
| --- | --- | --- | --- |
| 8 years ^43^ | Live-born preterm children with a birth weight <1750g (N=55) and controls (N=43) | N/A | A neurological examination and psychological assessments  for cases N= 42 (75%) and controls N=43, MRI for all cases |
| 16 years ^44^ | A subsample based on 16-year follow-up SWAN-screen, cases N=487 and controls N=315 | Psychiatric evaluation for cases N=268 (55.0%) and controls N=196 (62.2%) | Blood samplings |
| 18 years ^23^ | Cohort members living in the Oulu and surrounding municipalities N=2969 | Questions of history of low back pain history, medical history, quality of life, nutrition, socioeconomic status, leisure activities, history of injuries, occupational exposure, sports activities, smoking, and psychological factors (N=2012, 68%) | N/A |
| 19-22 years ^24^ | Subsample of the cohort members living in the Oulu and surrounding municipalities N=874 | N/A | MRI-scan of the lumbar spine (N=558, 64%) |
| 21-24-years ^44^ | Cases for risk of developing psychosis N=389 and their controls; psychotic episode N=78, ADHD N=103, random sample of the cohort N=193 | Background information, relationships, quality of life, substance use, Handedness, Coping.  Cases N=136 (35.0%) controls: psychotic episode N=27 (34.6%), ADHD N=52 (50.5%), random sample of the cohort N=80 (41.5%) | Several cognitive tests, psychiatric evaluation blood and urine sampling. Structural MRI |
| 24-years ^25^ | Preterm born subjects N=408 and controls N=579 | N/A | Blood samplings, Blood pressure, BMI, waist and hip measurements (N=378, 38.3%) |
| 25-27 years ^46^ | Cases based on exposure to maternal cigarette smoking (N=698) and their controls (N=698) | Background and lifestyle information, sleep questions, smoking, substance use and gambling, handedness, physical activity, diet, health and several psychological screens. Cases N=218, 31%; Controls N=253, 36% | Interview including SCID-I, family history of psychiatric disorders, psychiatric treatment history, Brief Nicotine Dependence Interview. Several cognitive tests, MRI of the brain, blood and urine samples. |
| 26 years ^26^ | Gynaecological health of young women N=4503 and their mothers | Questions on socio-demographic and other health background factors  mainly about reproduction, menstruation, and infertility (N=2770, 50%) | N/A |
| 29-32 years ^24^ | Subsample of those who attended sub-study of 19-22 years N=558 | N/A | MRI-scan of the lumbar spine (N=375, 67%) |
